# Impact of the COVID-19 Pandemic on Vascular Surgery Services in a United Kingdom Tertiary Center

**DOI:** 10.7759/cureus.95660

**Published:** 2025-10-29

**Authors:** Albagir Altahir, Mohammed Fageer, Lawrence Ugwumba, Peng Wong, Tamer M El-Sayed

**Affiliations:** 1 Vascular Surgery, James Cook University Hospital, Middlesbrough, GBR

**Keywords:** covid-19, emergency surgery, pandemic, service disruption, vascular surgery

## Abstract

Background: The COVID-19 pandemic significantly disrupted vascular surgery services worldwide. This study evaluates its impact on a UK tertiary vascular surgery center across successive lockdown phases.

Methods: A retrospective observational study compared vascular service activity across four time periods: pre-COVID-19 (P1; March 23-May 31, 2019), first lockdown (L1; March 23-May 31, 2020), second lockdown (L2; November 5-December 2, 2020), and third lockdown (L3; January 6-March 8, 2021).

Results: A total of 138 procedures were performed during P1, dropping to 42 in L1 and 35 in L2 (p < 0.03), before partially recovering to 86 in L3. Fourteen patients were COVID-19-positive, of whom 70% presented with arterial thrombosis. Aortic aneurysm repairs declined during L1 and L2 but rose significantly to 19 in L3. Thromboembolectomies doubled in all lockdowns compared to P1 (p < 0.05). Major amputations and emergency bypasses peaked during L3. Outpatient consultations fell sharply in L1 and L2 (p < 0.03), while telemedicine use increased tenfold by L3 (p < 0.05). All-cause mortality remained stable across all periods.

Conclusions: The COVID-19 pandemic profoundly affected elective vascular services while increasing emergency interventions. These findings highlight the need for resilient service structures and proactive strategies to maintain essential vascular care during future healthcare crises.

## Introduction

On March 11, 2020, the World Health Organization (WHO) declared COVID-19 a global pandemic, which has continued to challenge healthcare systems worldwide. By July 2021, over 180 million confirmed cases and more than four million deaths had been reported globally [1]. In the UK, over five million confirmed cases and more than 150,000 deaths were recorded [2,3]. The pandemic’s rapid spread and severity placed an overwhelming burden on healthcare systems across the world [4-6]. In the UK, the National Health Service (NHS) reported approximately 480,000 COVID-19-related hospital admissions [7]. To manage this pressure, NHS Foundation Trusts were instructed to prioritize urgent care, postponing or canceling non-urgent clinical work. Vascular surgery services were among those significantly affected. Organizing bodies such as the Vascular Society of Great Britain and Ireland (VSGBI) and the Society for Vascular Surgery issued guidance on managing vascular disease during the pandemic. These guidelines emphasized that most vascular patients are elderly, immunocompromised, and often have comorbidities such as ischemic heart disease or a smoking history, placing them at particularly high risk of severe COVID-19 infection [8].

To balance patient needs with hospital capacity, vascular services underwent unprecedented modifications. Following VSGBI recommendations in March 2020 [9], vascular procedures were triaged, with elective operations deferred except for those addressing immediately life- or limb-threatening conditions.

This study therefore aimed to evaluate the impact of the COVID-19 pandemic on vascular surgical service delivery in a UK tertiary center by comparing activity across three national lockdowns with an equivalent pre-pandemic period.

## Materials and methods

This was a retrospective observational study comparing vascular surgery services at James Cook University Hospital, Middlesbrough, a UK tertiary referral center, across four time periods: pre-COVID-19 (P1; March 23-May 31, 2019), the first UK lockdown (L1; March 23-May 31, 2020), the second lockdown (L2; November 5-December 2, 2020), and the third lockdown (L3; January 6-March 8, 2021). The number and type of procedures performed were recorded for each period. James Cook University Hospital is a high-volume vascular center providing elective and emergency care to a catchment population exceeding one million.

All patients who underwent vascular surgery or endovascular intervention during the defined periods were included, encompassing both elective and emergency cases. Outpatient activity, including face-to-face and telemedicine consultations, was also analyzed.

Data on inpatient and outpatient services were collected from electronic hospital records. Variables recorded included patient demographics (age, gender), surgical management of aortic aneurysms, lower limb revascularization, major lower limb amputation, renal vascular access, venous interventions, and extracranial cerebrovascular procedures. All-cause and COVID-19-related mortalities were compared between study periods.

Data analysis was performed using descriptive statistics. Comparisons between groups were conducted using Fisher’s exact and chi-square tests for categorical variables, and Student’s t-test or two-way ANOVA for continuous data. Analyses were performed using GraphPad Prism Version 8 (Dotmatics, Boston, MA, USA) and IBM SPSS Statistics Version 27.0 (IBM Corp., Armonk, NY, USA).

Ethical approval was not required for this service evaluation study, in accordance with UK Health Research Authority guidance, as all data were anonymized and collected as part of routine audit activity.

## Results

Inpatient services

**Figure 1:**
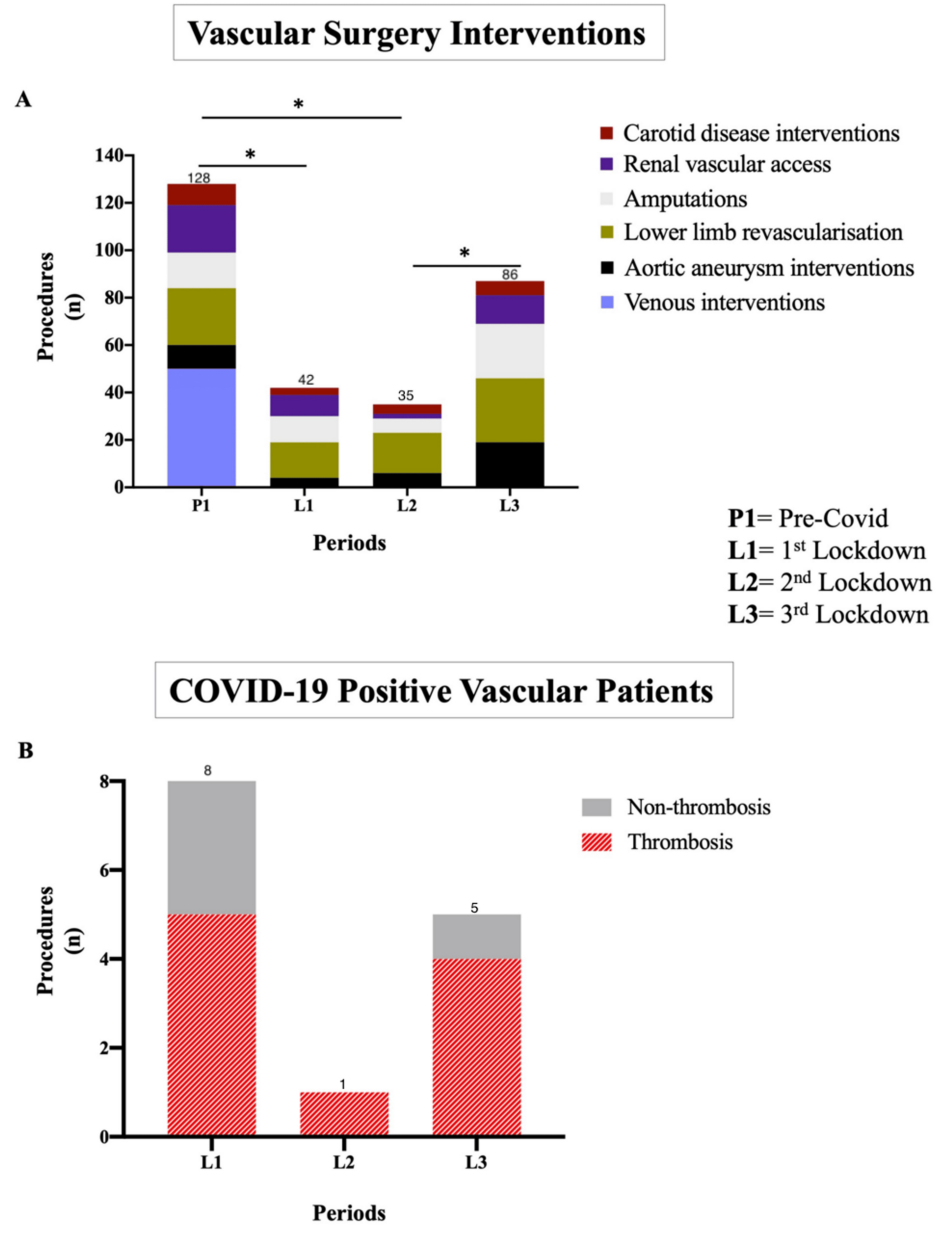
Vascular surgery interventions and COVID-19-positive vascular patients (A) Distribution of vascular interventions across the four study periods (P1, L1, L2, and L3). The number of interventions decreased sharply during L1 and L2 (p < 0.0001 for both, Wilcoxon matched-pairs signed-rank test) but recovered to 86 procedures during L3 (p < 0.0001). (B) Number of COVID-19-positive patients during the three UK lockdowns, with eight patients identified during the first pandemic wave; ten patients presented with COVID-19-related thrombosis. *p < 0.05.

The number of patients treated before COVID-19 dropped markedly from 138 (median age 64 years, 67.6% males) during P1 to 42 in L1 (median age 68 years, 69.4% males) and 35 in L2 (median age 72 years, 71.8% males) (p < 0.0001 for both, Wilcoxon matched-pairs signed-rank test). Activity partially recovered to 86 operations in L3 (p < 0.0001, Wilcoxon matched-pairs signed-rank test). Procedures were categorized into carotid interventions, renal vascular access, lower limb revascularization, major amputations, aortic aneurysm repairs, and venous interventions (Figure 1A).

Fourteen patients admitted to the vascular unit were found to be COVID-19-positive, with over half of them identified during the first pandemic wave. More than two-thirds of these patients tested positive for COVID-19 on admission and presented with COVID-19-related thrombosis (Figure 1B). Fewer than 25% (three patients) acquired infection during hospitalization, following negative swab results on admission, all during the first UK lockdown. Just over half of the COVID-19-positive group (nine patients) underwent emergency surgical interventions, while the remainder were managed conservatively. Among these patients, the limb loss rate was approximately 30%, and mortality was 14%.

Aortic Intervention

**Figure 2:**
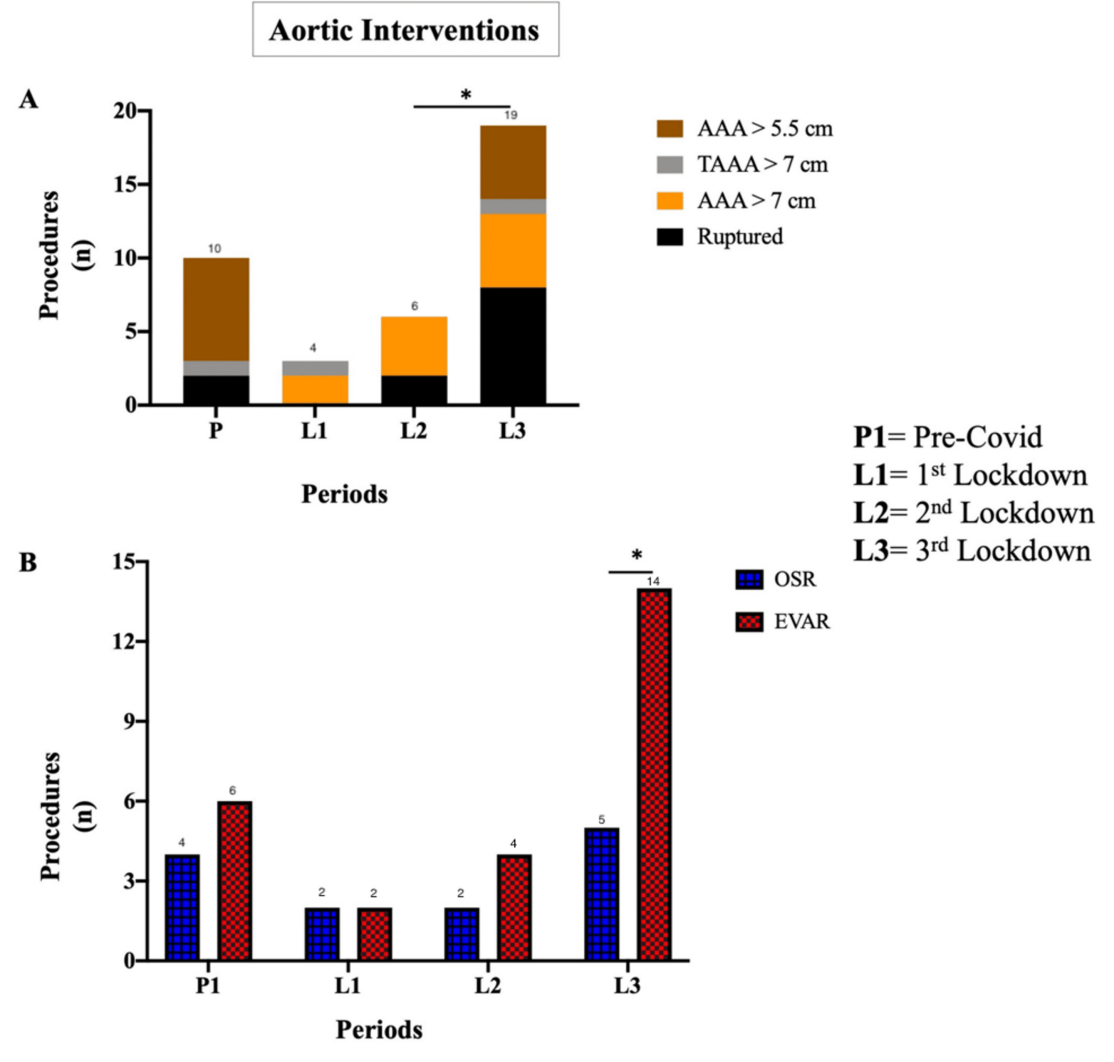
Aortic interventions during the COVID-19 pandemic (A) The number of aortic interventions declined during the first (from 10 to 4) and second lockdowns (6 repairs) but recovered in the third lockdown (19 procedures; p = 0.009). (B) Open and endovascular aortic aneurysm repairs were comparable before and during the first and second lockdowns, whereas in the third phase, EVAR was performed more frequently than open surgery (14 vs. 5 repairs; p = 0.04). AAA: abdominal aortic aneurysm, TAAA: thoraco-abdominal aortic aneurysm. *p < 0.05.

Aortic aneurysm interventions varied markedly across the four study periods. A total of 10 aortic aneurysm repairs (four open surgical repairs (OSRs) and six endovascular aneurysm repairs (EVARs)) were performed during P1, which dropped to four (two OSRs and two EVARs) in L1, and six (two OSRs and four EVARs) in L2 (p < 0.0001, Wilcoxon matched-pairs signed-rank test). Activity recovered to 19 repairs in L3 (p < 0.0001, Wilcoxon matched-pairs signed-rank test) (Figure 2A). During L3, EVARs significantly outnumbered OSRs (14 vs. 5 repairs; p = 0.0006, Wilcoxon matched-pairs signed-rank test) (Figure 2B).

Subgroup analysis of aortic interventions showed that the number of symptomatic or ruptured aneurysm repairs increased from two in P1 to eight in L3 (p < 0.0001, Wilcoxon matched-pairs signed rank test). A similar trend was observed for aneurysms measuring greater than 7 cm and 5.5 cm (p < 0.0001, Wilcoxon matched-pairs signed rank test). No significant changes were noted in thoraco-abdominal aneurysm repairs (Table 1).

**Table 1:** Aortic aneurysm repairs during the COVID-19 pandemic AAA: abdominal aortic aneurysm, TAAA: thoraco-abdominal aortic aneurysm.

Condition	P1 (n = 10)	L1 (n = 2)	L2 (n = 7)	L3 (n = 19)	p-value
Ruptured/symptomatic	2	0	2	8	<0.0001
AAA > 7 cm	0	2	4	5	<0.0001
TAAA > 7 cm	1	0	1	1	>0.05
AAA > 5.5 cm	7	0	0	5	<0.0001

Lower Limb Revascularization

**Figure 3:**
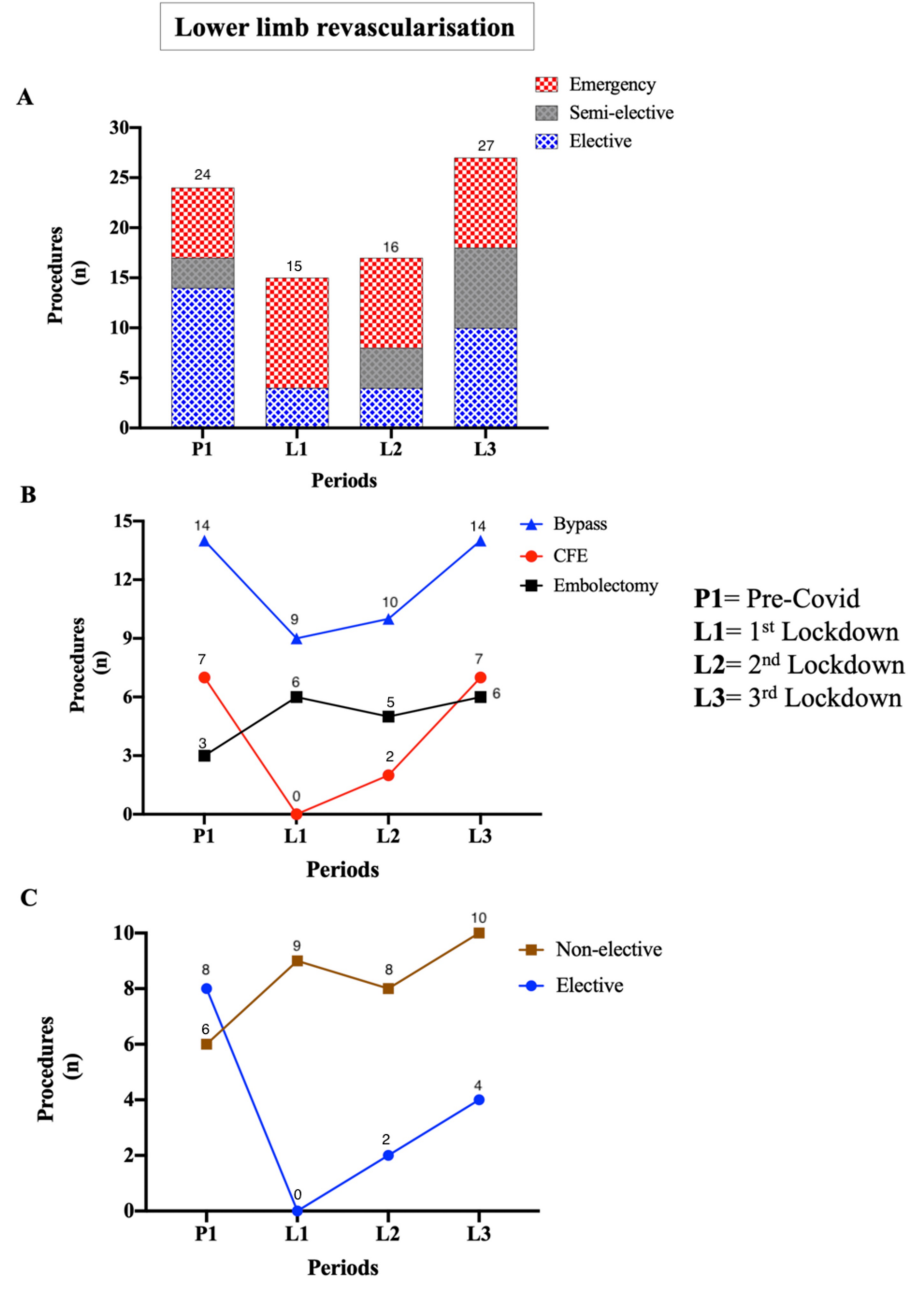
Lower limb revascularization before and during the COVID-19 pandemic (A) Total lower limb revascularization procedures declined from pre-COVID to the first lockdown (13 vs. 9) and recovered to 13 in the third lockdown. (B) Common femoral endarterectomies decreased during the first lockdown (p = 0.008) and gradually recovered in later phases, similar to lower limb bypass procedures, which dropped from 14 to 9 before recovering to 14 in L3. Emergency embolectomies doubled during the lockdown periods. (C) Non-elective bypass procedures increased during COVID-19 (6-10 operations), whereas elective procedures were temporarily halted in the first lockdown (p = 0.005) and partially recovered to four in L3. CFE: common femoral endarterectomy.

The total number of lower limb revascularization procedures fluctuated across the COVID-19 lockdown periods (p = 0.01). Procedures decreased from 13 pre-COVID to nine during the first lockdown (p = 0.05), then gradually recovered to 13 by the third lockdown (p = 0.05) (Figure 3A). Elective lower limb revascularizations fell markedly from seven pre-COVID to two and three in L2 and L3, respectively (p = 0.005). Conversely, semi-elective procedures increased from two in P1 to seven in L3 (p = 0.0002) (Table 2). The number of emergency limb salvage operations remained stable across all periods.

**Table 2:** Lower limb revascularization during the COVID-19 pandemic

	P1 (n = 13)	L1 (n = 9)	L2 (n = 10)	L3 (n = 13)	p-value
Elective	7	4	2	3	0.005
Semi-elective	2	0	4	7	0.0002
Emergency	4	5	4	3	>0.05

The types of lower limb revascularization procedures shifted notably during the COVID-19 pandemic. Common femoral endarterectomies for claudicants decreased sharply from seven in P1 to none in L1 (p < 0.0001) before gradually recovering to two in L2 and seven in L3 (p < 0.0001) (Figure 3B).

Emergency embolectomies doubled from three in P1 to seven in L1 (p = 0.03) and remained elevated in L2 (five procedures) and L3 (seven procedures) (p = 0.03), largely associated with COVID-19-related thrombosis.

Infra-inguinal bypass procedures decreased from 13 in P1 to 9 in L1 (p = 0.05), then recovered to 13 by L3 (p = 0.05) (Figure 3C). The number of emergency bypass operations doubled from 5 in L1 to 10 in L3 (p = 0.01), whereas elective bypasses approximately halved during L2 and L3 (p = 0.05 and p = 0.03, respectively) (Figure 3C).

Lower Limb Amputations

**Figure 4:**
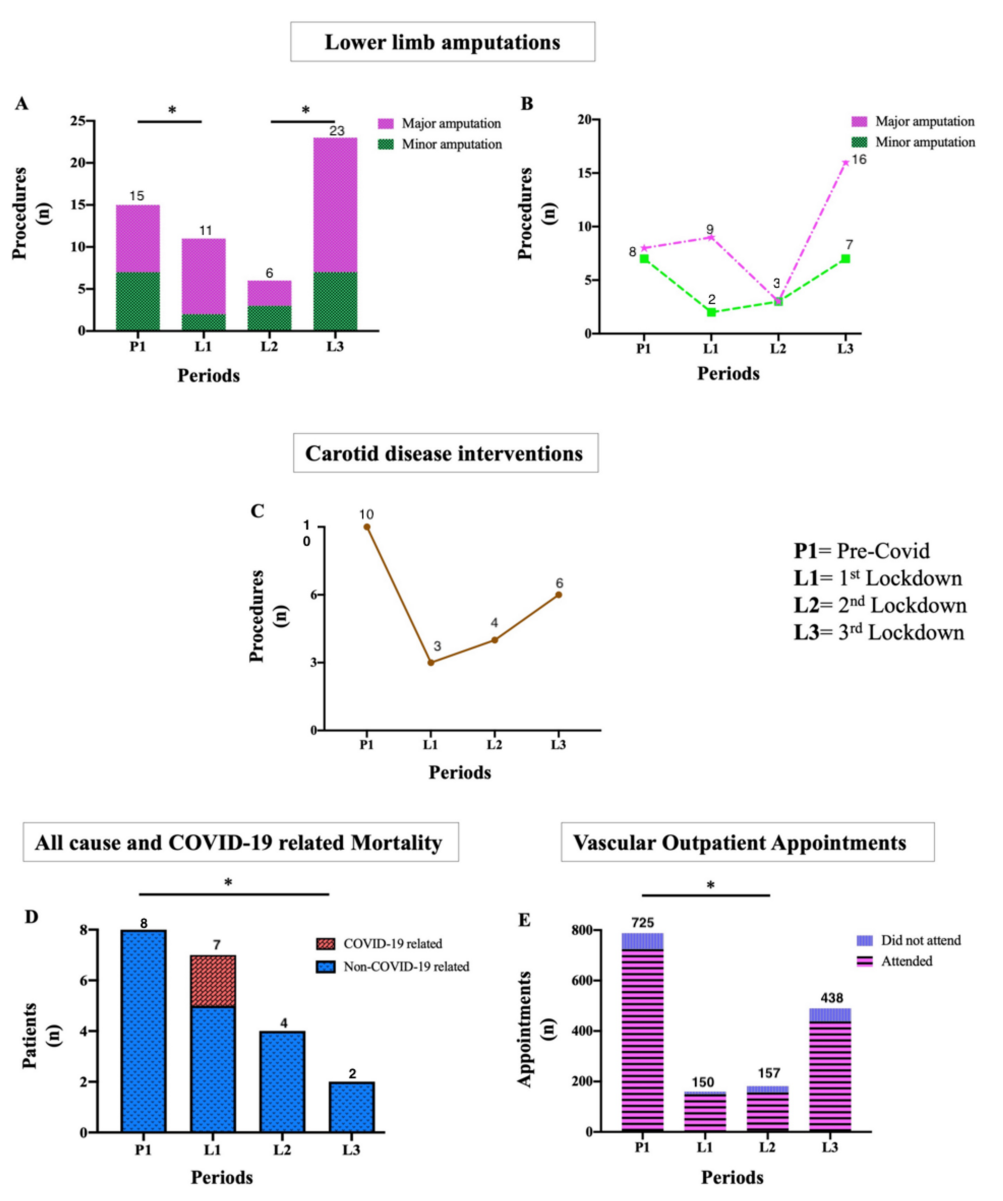
Lower limb amputations, carotid disease interventions, mortality, and outpatient consultations during COVID-19 (A) Lower limb amputations halved from P1 to L2 (15 vs. 6, p = 0.0008), then increased sharply in L3 (6 vs. 23, p < 0.0001). (B) Major and minor amputations both declined during early lockdowns and rose again in L3 (major: 8 in P1 vs. 16 in L3, p < 0.0001; minor: 3 in L2 vs. 7 in L3, p = 0.02). (C) Carotid endarterectomies dropped by one-third during the pandemic (10 in P1 vs. 3 in L1, p = 0.0003). (D) All-cause mortality remained stable throughout the pandemic (8 total deaths), including 2 COVID-19-related fatalities. (E) Telemedicine consultations increased markedly during the lockdowns, rising from 10 in P1 to 98 in L3 (p < 0.05). *p < 0.05.

Lower limb amputations nearly halved from P1 to L2 (15 vs. 6, p = 0.0008), then markedly increased from L2 to L3 (6 vs. 23, p < 0.0001) (Figure 4A).

Major amputations fluctuated throughout the COVID-19 period and eventually doubled compared to pre-pandemic levels (8 in P1 vs. 16 in L3, p < 0.0001). Minor amputations followed a similar trend, dropping during the first lockdown (7 in P1 to 2 in L1, p = 0.003) before recovering from L2 to L3 (3 in L2 to 7 in L3, p = 0.02) (Figure 4B).

Carotid Disease Intervention

Carotid endarterectomy for symptomatic extracranial cerebrovascular disease decreased by one-third during the first lockdown (10 in P1 to 3 in L1, p = 0.0003). The numbers gradually increased in L2 (four cases) and L3 (six cases), but this recovery did not reach statistical significance (Figure 4C).

Other Vascular Interventions

Varicose vein procedures were completely halted during the pandemic, compared to 50 interventions performed pre-COVID-19 (p < 0.0001). Renal arteriovenous (AV) access creation for hemodialysis fell sharply from 20 in P1 to 9 in L1 and 2 in L2 (p < 0.0001 for both), before partially recovering to 12 procedures in L3 (p < 0.0001).

Peri-COVID-19 Mortality

All-cause mortality remained relatively stable across the study periods: eight deaths pre-COVID-19, seven in L1 (including two COVID-19-related deaths), four in L2 (p = 0.05), and two in L3 (p = 0.001) (Figure 4D).

Outpatient services

Before COVID-19, 725 patients attended the vascular outpatient clinic. Attendance dropped by about three-quarters to 150 in the first lockdown and 157 in the second lockdown (p < 0.03 for both), then recovered to 438 in the third lockdown. Telemedicine consultations rose sharply during the pandemic, from 10 in P1 to 98 in L3 (p < 0.05) (Figure 4E).

## Discussion

Since the COVID-19 pandemic was declared in March 2020, the VSGBI, together with NHS England, Getting It Right First Time (GIRFT), and the Specialist Commission, has continuously updated its guidance as the situation evolved to ensure safe practice while maintaining life- and limb-saving services for vascular patients [9]. These essential services included treatment for symptomatic or ruptured AAA, acute and critical limb ischemia, symptomatic carotid artery disease, and revision or removal of non-functional or infected dialysis access. Consequently, vascular surgery practices underwent dramatic changes, with many centers focusing exclusively on urgent and emergency cases.

It was recognized early in the pandemic that the individuals most severely affected by COVID-19 were typically elderly males, smokers, and those with ischemic heart disease or immunocompromised states [10]. These risk factors are common among vascular patients. Therefore, the initial VSGBI guidance, and its subsequent updates, sought to balance these risks and support clinical decision-making when considering surgical intervention in this high-risk population [9]. Clinicians also needed to account for the strain on NHS Foundation Trust resources, including hospital bed capacity, length of stay, and critical care requirements [11].

In April 2020, during the first wave of the pandemic, our hospital implemented a COVID-19 outbreak management plan that divided the facility into two main zones: a COVID-19 (red) zone and a non-COVID-19 (green) zone. Only essential operations were performed on suspected or confirmed COVID-19 patients. Vascular emergencies, such as acute limb ischemia or ruptured aneurysms, were managed according to COVID-19 status. Patients with COVID-19 symptoms were directed to a designated COVID-19 CT scanner and operated on in the COVID-19 theater when indicated. Postoperatively, they were transferred to either the COVID-19 critical care unit or the COVID-19 ward (red zone). Non-COVID-19 patients, by contrast, utilized the general radiology CT scanner and non-COVID-19 emergency theater, then recovered in the non-COVID-19 critical care unit or ward (green zone). All inpatients were routinely swabbed on admission and re-tested if clinically indicated, such as upon symptom development or exposure to a COVID-19-positive patient. During the first lockdown, approximately half of the COVID-19-positive vascular patients acquired the infection during their hospital stay, despite initially testing negative by polymerase chain reaction (PCR) on admission.

The hospital’s COVID-19 management plan was revised in October 2020, introducing a three-zone system based on infection risk: red, amber, and green. The green zone was reserved for elective patients who had been isolated for 14 days before admission and tested COVID-19-negative within three days of admission. The amber zone was for patients with less than 14 days of isolation but a negative PCR test, while the red zone remained designated for confirmed COVID-19-positive patients. All inpatients were swabbed on days 3 and 5, and weekly thereafter. Patients testing positive at any stage were promptly transferred to the red zone. This revised system proved highly effective, as no vascular patients contracted COVID-19 during hospitalization in subsequent lockdowns. This improvement reflected enhanced understanding of viral transmission, better infection control practices, increased awareness among staff and patients, and the greater availability of personal protective equipment.

In April 2021, the hospital revised its zoning system into two pathways, blue and red, based on triage screening. COVID-19-negative patients were admitted to the standard (blue) pathway, while COVID-19-positive patients were managed through the red pathway. Consistent with findings from the second lockdown, no vascular inpatients contracted COVID-19 during this period, underscoring the effectiveness of the hospital’s infection prevention strategy.

Regarding aortic aneurysm management, the VSGBI initially recommended increasing the threshold for elective aneurysm repair from 5.5 cm to greater than 7 cm, or to proceed only with symptomatic aneurysms deemed at imminent risk of rupture. Similarly, the American College of Surgeons’ COVID-19 Guidelines suggested intervention for aneurysms measuring 6.5 cm or larger, a position supported by an international survey in which over 10% of vascular centers reported repairing aneurysms exceeding 6.5 cm [12].

These recommendations were reflected in our findings, which showed a reduction in aortic aneurysm repairs during the first and second lockdowns. The deferral of elective repair for aneurysms between 5.5 and 6 cm, coupled with the temporary suspension of the UK Abdominal Aortic Aneurysm (AAA) Screening Programme, led to a growing backlog of patients awaiting intervention [13]. Consequently, AAA repairs tripled during the third lockdown, with EVAR performed more frequently than open surgical repair, following VSGBI guidance to minimize critical care utilization. Open surgery was reserved for cases unsuitable for EVAR but with a high likelihood of success, taking into account intensive care unit capacity before proceeding. Notably, the number of ruptured AAAs increased from two in P1 to eight in L3, which may reflect delayed elective repair, suspension of surveillance programs, or patient hesitancy to seek medical care due to fear of COVID-19 exposure.

The management of peripheral arterial disease evolved throughout the COVID-19 pandemic, influenced by continually updated national guidance [9]. Urgent intervention was reserved for immediately threatened limbs, which was reflected in our findings: a sharp decline in elective lower limb revascularization during the first and second lockdowns, followed by a gradual recovery by the third lockdown.

This pattern likely reflects a more conservative treatment approach during the initial pandemic phases, driven by limited hospital resources, particularly critical care bed availability. As experience in managing COVID-19 improved and operational pressures eased, the vascular service became increasingly interventional, allowing revascularization activity to return to near pre-pandemic levels during L3.

Conversely, emergency procedures such as lower limb bypasses and embolectomies doubled during the lockdowns, corresponding with an increase in critical and acute limb ischemia, likely linked to COVID-19-associated arterial thrombosis. Approximately 70% of COVID-19-positive vascular patients in our series presented with acute thrombosis, consistent with findings from other UK tertiary vascular centers [14,15]. Mestres et al. similarly reported a marked rise in arterial thrombosis as a complication of COVID-19 infection [16]. Patients with non-limb-threatening ischemia were diverted to a dedicated “hot” vascular clinic for further evaluation. Consequently, elective procedures such as common femoral endarterectomy for claudicants were halted during the first lockdown, and elective bypass operations were reduced by approximately half throughout the pandemic.

Alongside the increase in emergency bypass surgery and embolectomy, our center also observed a significant rise in amputation rates. This may partly reflect the early VSGBI guidance, which recommended primary amputation in selected patients instead of complex revascularizations or repeated debridements, to minimize hospital stay and reduce strain on healthcare resources.

We observed that the emergency amputation rate initially halved during the first lockdown, likely reflecting a more conservative approach to limb salvage. However, by the third lockdown, the rate had quadrupled, suggesting that the increase in amputations may have resulted from disease progression in patients whose revascularization was deferred or no longer feasible due to delayed presentation.

The management of carotid artery disease during the pandemic required careful consideration of both surgical risks and available resources. The VSGBI emphasized that the need for postoperative critical care beds and the heightened COVID-19 risk among vascular patients should guide clinical decisions. As a result, carotid endarterectomy was recommended only for patients with crescendo transient ischemic attacks (TIAs) or those at high risk of major stroke.

Consequently, the time from referral to procedure increased, with many patients undergoing surgery beyond the recommended two-week window from their initial cerebrovascular event. This challenge was not unique to our center. The World Stroke Organization (WSO) reported a global reduction in acute stroke services during the pandemic, including the scaling down or suspension of endovascular procedures, and widespread postponement of CEA due to critical care bed shortages [17].

Renal AV access formation services also slowed but were not completely halted, as maintaining dialysis access was vital for patient survival. The vascular and renal teams collaborated closely to prioritize urgent AV access cases throughout the first two pandemic waves, with services beginning to recover during the third lockdown as hospital capacity stabilized and elective procedures resumed.

Non-urgent vascular interventions, including varicose vein procedures, were suspended during the pandemic, resulting in a backlog of patients with symptomatic or complicated venous disease awaiting treatment. Likewise, elective arterial surgeries, such as those for asymptomatic carotid stenosis, chronic limb ischemia (claudicants), and asymptomatic or small aneurysms below the intervention threshold of 7 cm, were deferred in accordance with national guidance. The long-term consequences of postponing these so-called “elective” vascular procedures remain uncertain and warrant future evaluation.

Importantly, there was no increase in all-cause mortality during the pandemic compared to the pre-COVID-19 period, and only two COVID-19-related deaths were recorded. This outcome likely reflects the effectiveness of the hospital’s conservative management strategy, which minimized inpatient admissions, shortened hospital stays, and prioritized life- or limb-saving interventions.

Routine outpatient clinics were also temporarily deferred, with only urgent cases reviewed either face-to-face (when necessary) or virtually via telephone. During the first lockdown, outpatient capacity was reduced to approximately 25% of normal levels, consistent with reports from other UK vascular centers, which documented similar reductions of around 30% [12]. Our center introduced daily urgent clinic slots for community referrals with suspected life- or limb-threatening vascular disease. Referrals from primary care were triaged by the on-call consultant under the Referral Assessment Service, resulting in one of three outcomes: “accept but defer,” “accept and see,” or “telephone advice and return to primary care.” To minimize hospital visits, post-discharge follow-up was limited to telephone contact on an as-needed basis. The adoption of telephone consultations and telemedicine significantly expanded outpatient capacity by approximately tenfold during the second lockdown (L2). Combined with the implementation of vascular hot clinics for acute and urgent patients, this innovation enabled the service to triple its outpatient throughput during the third lockdown compared to the first.

Looking forward, it will be crucial to evaluate how the shift toward urgent and emergent care during the pandemic has affected long-term outcomes for vascular patients. Understanding whether deferred cases have progressed beyond the point of safe or effective intervention will be essential for future service planning [9].

This study has several limitations. It is a retrospective, single-center analysis with a relatively small sample size, which limits the generalizability of findings. Additionally, the independent effect of COVID-19 is difficult to isolate from other confounding factors that may have influenced vascular service delivery and outcomes.

## Conclusions

COVID-19 has had a profound impact on vascular surgery services worldwide, including in the UK. Managing vascular disease during the pandemic necessitated significant deviations from conventional pathways, further complicated by the emergence of COVID-19-related vascular emergencies. Balancing the risks and benefits of interventions while accounting for critical care bed availability and the overall strain on healthcare systems posed unprecedented challenges. Despite these obstacles, clinicians, specialty societies, and healthcare institutions adapted rapidly to maintain patient safety and continuity of service. This study highlights how our institution responded to these challenges and adapted service delivery to ensure the safe management of vascular patients. The insights gained provide valuable guidance for improving the preparedness and resilience of healthcare systems in the face of future pandemics and other unforeseen crises.
